# Functional Connectivity Correlates of Perceived Financial Exploitation in Older Adults

**DOI:** 10.3389/fnagi.2020.583433

**Published:** 2020-11-12

**Authors:** Gali H. Weissberger, Laura Mosqueda, Annie L. Nguyen, Jenna Axelrod, Caroline P. Nguyen, Patricia A. Boyle, Nathan Spreng, S. Duke Han

**Affiliations:** ^1^Department of Family Medicine, USC Keck School of Medicine, Alhambra, CA, United States; ^2^Interdisciplinary Department of Social Sciences, Bar-Ilan University, Ramat Gan, Israel; ^3^USC School of Gerontology, Los Angeles, CA, United States; ^4^Rush Alzheimer’s Disease Center, Rush University Medical Center, Chicago, IL, United States; ^5^Department of Behavioral Sciences and Psychiatry, Rush University Medical Center, Chicago, IL, United States; ^6^Department of Neurology and Neurosurgery, McGill University, Montreal, QC, Canada; ^7^Departments of Psychiatry and Psychology, McGill University, Montreal, QC, Canada; ^8^McConnell Brain Imaging Centre, Montreal Neurological Institute, McGill University, Montreal, QC, Canada; ^9^Department of Psychology, USC Dornsife College of Letters, Arts, and Sciences, Los Angeles, CA, United States; ^10^Department of Neurology, USC Keck School of Medicine, Los Angeles, CA, United States

**Keywords:** financial exploitation, older adults, resting-state fMRI, functional connectivity, hippocampus, insula, medial frontal cortex, decision making

## Abstract

Financial exploitation (FE) in old age is devastating and common; however, the neural correlates of FE are poorly understood. Previous studies of FE in older adults have implicated declines in decision making and social cognition as two risk factors for FE in later life. Here we examined whether functional connectivity among brain regions implicated in decision making and social cognition differed for those with an experience of FE vs. those without. Participants included 16 older adults without cognitive impairment who reported FE (Mean age = 70.5, 62.5% female, Mean education = 16.0 years) and 16 demographically and cognitively matched adults who denied a history of FE (Mean age = 65.1, 37.5% female, Mean education = 15.1 years). Measures of whole-brain resting-state functional connectivity in the hippocampus, insula, and medial frontal cortex were derived for each group. Compared to the non-FE group, FE was associated with greater functional connectivity between the right hippocampus and bilateral temporal regions, and less functional connectivity between the right hippocampus and the right cerebellum and bilateral lingual gyri. The FE group showed less connectivity between the right and left insula and cingulate cortex, and between the right insula and regions of the left lateral temporal gyrus and dorsolateral prefrontal cortex. Finally, the FE group showed greater functional connectivity between the medial frontal cortex and the right lateral temporal gyrus and orbitofrontal cortex, and less functional connectivity with the right pre- and postcentral gyri. Results suggest that perceived FE in old age is associated with whole-brain functional connectivity differences involving the hippocampus, insula, and medial frontal cortex, consistent with models implicating age-associated changes in decision making and social cognition in FE.

## Introduction

Financial exploitation (FE) is one of the most commonly reported types of elder abuse (Amstadter et al., 2011; Lachs and Berman, 2011). It is estimated that older adults lose 3 billion (MetLife, 2011) to 36 billion (True Link Financial, 2015) dollars annually to FE, including both scams and fraud, in the United States. The experience of FE can be devastating to older adults, many of whom live on fixed incomes due to retirement and thus cannot regain money lost through additional earnings in the workplace (Nerenberg, 1999). While cognitive impairment is a known correlate of FE (Wilber and Reynolds, 1997; Choi et al., 1999; Dong et al., 2011; Gamble et al., 2014; James et al., 2014; Wood et al., 2014; Han et al., 2016a; Lichtenberg et al., 2016; Boyle et al., 2019), reports of FE among cognitively intact older adults are common (Templeton and Kirkman, 2007; Jackson and Hafemeister, 2011). Despite the significant personal, familial, and societal impact of FE, little is known about the underlying neural correlates of FE in older adults.

The cognitive correlates of FE in older adulthood, while still being elucidated, could inform our understanding of underlying brain differences that signal elevated FE risk. Potential mechanisms may involve age-related changes in decision making (Boyle et al., 2012a; Spreng et al., 2016) and social-cognitive processes (Moran et al., 2012; Mather, 2016; Spreng et al., 2017). Deficits in decision making have been demonstrated even in cognitively healthy older adults (Denburg et al., 2005; Boyle et al., 2012b; Tymula et al., 2013; Spreng et al., 2016; Bangma et al., 2017), with studies documenting less advantageous decisions in older adults relative to young adults (e.g., Denburg et al., 2005). These disadvantageous decisions may in part be driven by several age-related cognitive and affective changes that have been well-documented in the literature. For example, older adults demonstrate a positivity bias in which they show memory and attention preferences towards positively valenced stimuli over negatively valenced stimuli (Mather, 2016). This bias is thought to impact the decision-making strategies of older adults by affecting the weight placed on the costs and benefits of decisions (Samanez-Larkin and Knutson, 2015). For example, older adults exhibit less negative effects when anticipating loss compared to young adults (Nielsen et al., 2008). Decision making also involves prospection, or the representation of future outcomes, to simulate how events will unfold following a decision (Weierich et al., 2011). This ability is thought to be more challenging for older adults due to age-related neuropathological changes to brain regions important for prospection, and its decline may negatively impact decision making, increasing susceptibility to scams (Han et al., 2013, 2016b; Lamar et al., 2019). Finally, evidence suggests that older adults are more trusting of others (Castle et al., 2012) and exhibit a positivity bias when forming impressions of others (Cassidy et al., 2013). These age-related changes in social cognition could also contribute to greater risk of FE.

Functional connections between brain regions involved in decision making and social cognition may thus contribute to increased FE risk in older adults. A growing body of research has implicated a network of brain regions as important for economic decision making and social cognition, notably including the medial prefrontal cortex (mPFC), medial temporal lobes, and the insular cortex. The mPFC is the anterior hub of the default network (Raichle, 2015) that is implicated in emotional decision making and prosocial behavior (Mather, 2016). It has been associated with tasks of decision making that involve immediate and delayed rewards (McClure et al., 2004; Peters and Buchel, 2011; Han et al., 2013) and risk-taking (Tom et al., 2007; Yu et al., 2017). The mPFC has been specifically implicated in social impression formation, with one study showing that older adults recruited the region more when forming positive impressions compared to young adults who recruited it more when forming negative impressions (Cassidy et al., 2013). The medial temporal lobes, including the hippocampus and parahippocampal gyri, have been implicated in studies of risk aversion (Han et al., 2012) and temporal discounting (preference for smaller immediate rewards vs. larger delayed rewards; Han et al., 2013), possibly reflecting their involvement in prospective imagery (Peters and Buchel, 2011). Our group has also found this region to be particularly implicated in studies of susceptibility to scams in older adults (Han et al., 2016b; Lamar et al., 2019). The insular cortex has been cited for its role in interpersonal trust and trust evaluations (Castle et al., 2012; Belfi et al., 2015), decision making during risk and uncertainty (Singer et al., 2009; Mather, 2016), and temporal discounting (Wittmann et al., 2010). A study by Castle et al. (2012) found that older adults rated faces with untrustworthy cues as more trustworthy compared to young adults, and showed muted activation in the anterior insula when shown untrustworthy faces. Using a data-driven independent components analysis (ICA) approach, Spreng et al. (2017) were the first to investigate neural correlates of FE. They reported cortical thinning in the anterior insula and posterior superior temporal cortices, regions associated with processing affective and social information. Further, connectivity within the salience network (e.g., bilateral anterior insula, anterior cingulate cortex; Seeley et al., 2007) and default network (e.g., medial prefrontal cortex, posterior cingulate cortex, inferior parietal lobule, lateral and medial temporal lobes; Andrews-Hanna et al., 2014; Raichle, 2015) was reduced, while between network connectivity (specifically between default network regions and the anterior insula) was increased in older adults who self-reported a history of FE (Spreng et al., 2017). Taken together, neural changes to these regions may contribute to poor decision making and consequently increased risk of FE.

In the present study, we examined whole-brain, voxelwise functional connectivity differences for three regions of interest between a diverse group of cognitively healthy older adults who self-reported a history of FE (FE group) and demographically-matched older adults who denied a history of FE (non-FE group) participating in the Finance, Cognition, and Health in Elders Study (FINCHES). We hypothesized differences in functional connectivity profiles for the medial frontal cortex, hippocampus, and insula between FE and non-FE groups, providing support for existing models implicating decision-making and social cognition in FE risk in older adults (Han et al., 2013, 2016b; Spreng et al., 2017; Lamar et al., 2019).

## Materials and Methods

### Participants

Thirty-five older adults aged 50 or older underwent magnetic resonance imaging (MRI) as part of their participation in the pilot phase of the Finance, Cognition, and Health in Elders Study (FINCHES). To be eligible for study enrollment, participants had to have no known signs of cognitive impairment or a diagnosis of dementia, a neurological or psychiatric illness, or current problems with drugs or alcohol, as assessed *via* telephone screen. On the day of their evaluation, participants were further screened for significant cognitive impairment using the Montreal Cognitive Assessment (MoCA) screening instrument. Per protocol as of the date of this submission, participants who score 23 or below on the MoCA (Carson et al., 2018) were ineligible for further study participation. Three participants were excluded from the present analyses, leaving a total sample of 32 participants. One of these three participants reported a past long-standing history of drug abuse towards the end of participation in the study. A second participant was excluded due to inconsistent information reported to study examiners. A third participant was excluded due to a history of hydrocephalus.

Before undergoing the MRI, participants completed a series of behavioral and cognitive measures. All study procedures were approved by the institutional review board of the University of Southern California, and consent to participate was provided by all participants, following institutional review board guidelines.

### Perceived Financial Exploitation

To assess the history of perceived financial exploitation (FE), participants were asked two questions: (1) “Do you feel you have been taken advantage of financially?” and (2) “Does someone you know feel that you have been taken advantage of financially?” Participants who provided an affirmative response to either question were included in the perceived FE group. Those who responded “no” to both questions were placed in the non-financially exploited (non-FE) group. The specific questions chosen are consistent with previously published work (Gamble et al., 2013, 2014; Lichtenberg et al., 2016; Weissberger et al., 2020) and were chosen because they capture a wide range of perceived FE experiences.

Sixteen of the 32 (50.0%) participants were included in the perceived FE group. Of these, one individual answered no to “Do you feel you have been taken advantage of financially?” but yes to “Does someone you know feel that you have been taken advantage of financially?” Seven participants responded yes to both questions, and seven responded yes to only the first question (“Do you feel you have been taken advantage of financially?”). One participant did not complete the questionnaire but made it clear to study staff that they perceived experience of FE during study participation (i.e., during the telephone screen), and was thus included in the perceived FE group. Sixteen participants responded “no” to both questions and were thus included in the non-FE group.

### Neuroimaging

Brain MRI scans were conducted with a Siemens Magnetom Prisma 3 Tesla MRI scanner. A high resolution T1-weighted anatomical scan was acquired using a 3D magnetization-prepared rapid acquisition gradient-echo (MPRAGE) sequence with a time echo (TE) of 2.95 ms, repetition time (TR) of 2.3 s, flip-angle = 9°, field-of-view (FOV) read = 270 mm, FOV phase = 93.8%, 176 sagittal slices, slice thickness = 1.2 mm, voxel size 1.1 mm × 1.1 mm × 1.2 mm, 240 × 256 acquisition matrix. Resting-state MRI data were acquired using an echo-planar imaging (EPI) sequence with a TR = 607 ms, TE = 32 ms, flip angle = 50°, matrix size = 88 mm × 88 mm, 64 axial slices, 2.5 mm slice thickness, the field of view (FOV) read = 220 mm, FOV phase = 100%, voxel size = 2.5 × 2.5 × 2.5 mm^3^, a multiband factor of 8, and 946 acquisitions with interleaved slice ordering for a total of 9 mins and 42 sper scan. Participants were instructed to keep their eyes open.

Image processing and analyses were conducted in MATLAB using the CONN Toolbox version 18.a^1^ (Whitfield-Gabrieli and Nieto-Castanon, 2012). The CONN toolbox’s default pipeline was utilized for preprocessing and quality assurance of structural and functional scans. First, functional scans underwent realignment and slice-timing correction. Next, functional and structural coregistration occurred. Then spatial normalization was implemented, followed by smoothing using a full-width half-maximum (FWHM) isotropic Gaussian kernel filter of 8 mm. Structural images were segmented anatomically according to gray matter, white matter, and cerebrospinal fluid maps, and functional and structural images were normalized to Montreal Neurological Institute (MNI) space (MNI152). As part of the preprocessing pipeline and before normalization and smoothing, the Artifact Detection Toolbox (ART^2^) was used to identify artifact and motion outliers according to conservative settings (95^th^ percentile of the normative sample). Time points were identified as outliers if the global mean signal intensity exceeded 3 standard deviations or if moving from a preceding image exceeded a 0.5 mm deviation. These time points were included as regressors in addition to 12 head motion parameters derived from the realignment step and principal components delineated from white matter and cerebrospinal fluid (10 components for white matter, five components for cerebrospinal fluid) during a denoising step (nuisance signal regression) that occurred after smoothing. Finally, a band-pass filter was applied to the functional data with a frequency window of 0.008–0.09 Hz (Whitfield-Gabrieli and Nieto-Castanon, 2012; Nieto-Castanon, 2020).

### Statistical Analyses

A voxelwise whole-brain to the region of interest (ROI) analysis approach was taken. Anatomically-derived ROIs were defined according to the Harvard-Oxford Brain Atlas, per the CONN Toolbox protocol (Whitfield-Gabrieli and Nieto-Castanon, 2012). These included the medial frontal cortex, left and right hippocampus, and left and right insula, for a total of five separate ROIs. For individual-level analyses, a mean signal time course for each ROI was calculated, and Pearson correlations between the ROI signal time course and the time series of every other voxel in the brain were calculated. Correlation coefficients were converted to normally distributed standardized scores using Fisher’s z-transformation. Subject-specific connectivity maps for each ROI were then used in second-level General Linear Model analyses to examine differences in functional connectivity between FE and non-FE groups for each* a priori* specified ROI. Age, education, sex, and global cognition (total scores on the MoCA) were included as covariates in the models. Individual voxels were considered significant at a *p*-value < 0.005 and clusters of voxels were considered significant at a false discovery rate (FDR)-corrected threshold of *p* < 0.05, two-tailed. We selected two-tailed tests given* a priori* hypothesis that group differences in functional connectivity could arise in either direction across the ROIs under investigation.

## Results

### Participant Characteristics

Participant demographics are presented in Table 1. Groups did not differ on age, education, sex, race/ethnicity, and total score on the MoCA (all *p-*values ≥ 0.16). Regarding race and ethnicity breakdown, 11 perceived FE and eight non-FE were non-Hispanic White, one perceived FE and four non-FE were African American, three perceived FE and three non-FE were Asian, and one perceived FE and one non-FE self-reported Hispanic ethnicity but chose not to indicate race.

**Table 1:** Sample characteristics of FE (*n* = 16) and non-FE groups (*n* = 16).

	FE (*n* = 16)		Non-FE (*n* = 16)		
	M (range)	SD	M (range)	SD	*p*-value*
Age	70.50 (53–93)	12.97	65.13 (51–76)	8.48	0.176
Education	16.00 (12–20)	2.53	15.06 (11–20)	3.00	0.347
Sex (%female)	62.5%	-	37.5%	-	0.157
MoCA	27.69 (26–30)	1.40	27.63 (26–30)	1.45	0.902
Race (%Non-Hispanic White)	68.8%	-	50.0%	-	0.280

Note: FE, financially exploited; M, mean, SD, standard deviation; *p-values reflect the results of two-sample independent *t*-tests or Fisher’s Exact Tests comparing FE and non-FE groups.

### Hippocampal Functional Connectivity

**Figure 1:**
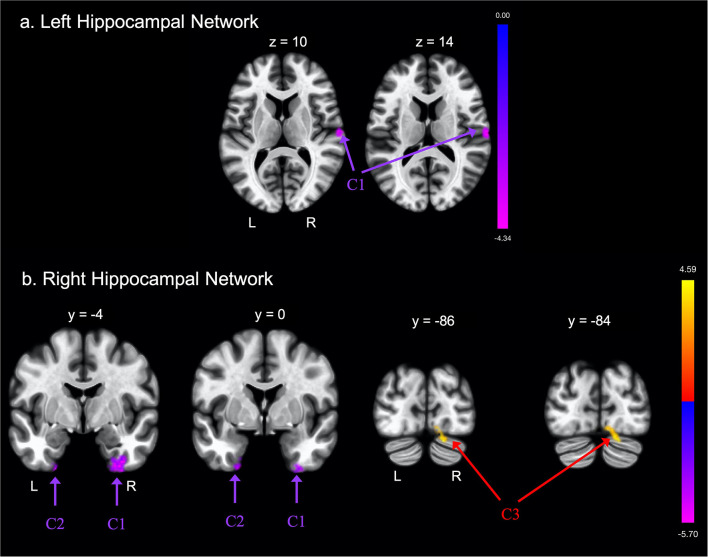
Group differences in functional connectivity with the **(A)** left hippocampus and **(B)** right hippocampus regions of interest (ROIs), after adjusting for age, education, sex, and global cognition. Clusters corrected for multiple comparisons using a false discovery rate (FDR)-corrected threshold of *p* < 0.05 (voxel *p* < 0.005), two-tailed. Values shown in the color bar correspond to t-scores. Negative (blue) clusters reflect those with significantly greater connectivity to the ROI in perceived Financial Exploitation (FE) compared to non-FE older adults; positive (red) clusters reflect those with significantly less connectivity to the ROI in perceived FE compared to non-FE older adults.

Groups differed concerning functional connectivity of the left and right hippocampus (Tables s 2A,B and Figures 1A,B, respectively). After adjusting for covariates including age, sex, education, and MoCA scores, FE was associated with lower functional connectivity between the right hippocampus and one cluster that included portions of the right cerebellum crus, bilateral lingual gyri, and right occipital fusiform gyrus.

**Table 2:** FE (*n* = 16) and non-FE (*n* = 16) group differences in functional connectivity with the **(A)** left hippocampus, **(B)** right hippocampus, **(C)** left insula, **(D)** right insula, and **(E)** medial frontal cortex seed regions, after adjusting for age, education, sex, and scores on the Montreal Cognitive Assessment (MoCA).

Seed ROI	Cluster	Regions	MNI coordinates			Cluster size	*t*-value^1^
			*x*	*y*	*z*		
(a) Left hippocampus	1	Right postcentral gyrus	66	−18	14	215	−4.18
		Right central opercular cortex
		Right planum temporale
(b) Right hippocampus	1	Left temporal pole	−22	0	−48	340	−4.98
		Left temporal fusiform cortex
		Left parahippocampal gyrus
	2	Right temporal fusiform cortex	32	−4	−46	292	−4.78
		Right parahippocampal gyrus
		Right inferior temporal gyrus
	3	Right cerebellum crus	16	−86	−24	213	5.85
		Bilateral lingual gyri
		Right occipital fusiform gyrus
(c) Left insula	1	Anterior cingulate gyrus	6	40	20	224	5.53
		Bilateral paracingulate gyri
	2	Right frontal orbital cortex	38	32	−18	195	5.56
		Right frontal pole
	3	Posterior cingulate gyrus	−4	−26	40	135	5.20
		Anterior cingulate gyrus
(d) Right insula	1	Anterior cingulate gyrus	6	8	36	1218	5.42
		Bilateral paracingulate gyri
	2	Left middle temporal gyrus	−70	−22	−10	269	4.82
		Left superior temporal gyrus
		Left temporal pole
	3	Left dorsolateral prefrontal cortex	−40	46	26	153	4.43
	4	Posterior cingulate gyrus	−4	−22	38	106	5.22
		Anterior cingulate gyrus
(e) Medial frontal cortex	1	Right middle temporal gyrus	58	−4	−8	841	−5.17
		Right superior temporal gyrus
	2	Right temporal pole	40	26	−24	182	−4.98
		Right orbitofrontal cortex
	3	Right postcentral gyrus	44	−6	14	136	4.12
		Right precentral gyrus
		Right central opercular cortex

*Note: MNI, Montreal Neurological Institute; ^1^Negative *t*-values reflect regions of greater functional connectivity in the FE group than the non-FE group*.

FE was associated with greater functional connectivity between the left hippocampus and one cluster that included the right postcentral gyrus, right central opercular cortex, and right planum temporale. FE was associated with greater functional connectivity between the right hippocampus and two clusters. The first cluster included portions of the left temporal pole, left fusiform gyrus, and left parahippocampal gyrus; the second cluster included portions of the right fusiform gyrus, right parahippocampal gyrus, and the right inferior temporal gyrus.

### Insular Functional Connectivity

**Figure 2:**
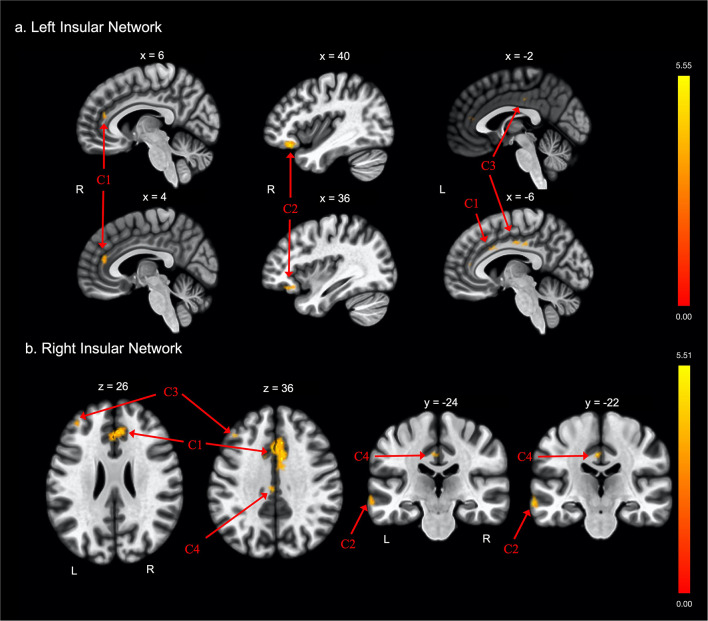
Group differences in functional connectivity with the **(A)** left insula and **(B)** right insula ROIs, after adjusting for age, education, sex, and global cognition. Clusters corrected for multiple comparisons using a false discovery rate (FDR)-corrected threshold of *p* < 0.05 (voxel *p* < 0.005), two-tailed. Values shown in the color bar correspond to t-scores. Negative (blue) clusters reflect those with significantly greater connectivity to the ROI in perceived FE compared to non-FE older adults; positive (red) clusters reflect those with significantly less connectivity to the ROI in perceived FE compared to non-FE older adults.

There were also differences between groups in functional connectivity of the left and right insular cortex (Tables s 2C,D and Figures 2A,B, respectively). The FE group exhibited lower functional connectivity than the non-FE group between the left insula and three clusters. The first cluster spanned the anterior cingulate gyrus and bilateral paracingulate gyri. The second cluster included the right frontal orbital cortex and right frontal pole. The third cluster included the anterior and posterior cingulate gyri. The FE group exhibited less functional connectivity than the non-FE group between the right insula and four clusters. The first cluster included portions of the anterior cingulate gyrus and bilateral paracingulate gyri. The second cluster included portions of the left middle and superior temporal gyri, and the left temporal pole. The third cluster included a portion of the dorsolateral prefrontal cortex. The fourth cluster included the anterior and posterior cingulate gyrus.

There were no findings of greater functional connectivity between the left and right insula and other brain regions in the FE group compared to the non-FE group.

### Medial Frontal Cortex Functional Connectivity

**Figure 3:**
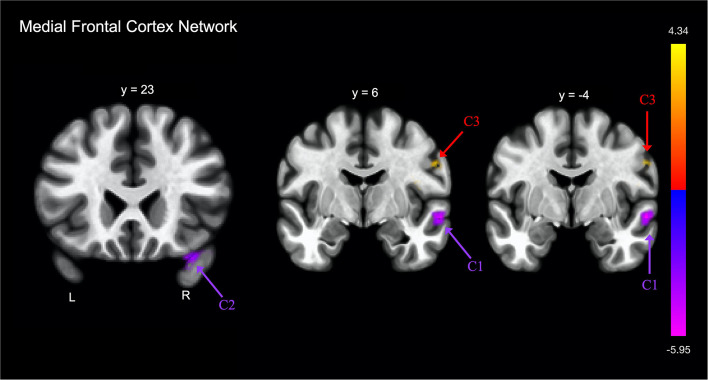
Group differences in functional connectivity with the medial frontal cortex, after adjusting for age, education, sex, and global cognition. Clusters corrected for multiple comparisons using a false discovery rate (FDR)-corrected threshold of *p* < 0.05 (voxel *p* < 0.005), two-tailed. Values shown in the color bar correspond to t-scores. Negative (blue) clusters reflect those with significantly greater connectivity to the ROI in perceived FE compared to non-FE older adults; positive (red) clusters reflect those with significantly less connectivity to the ROI in perceived FE compared to non-FE older adults.

FE was associated with lower functional connectivity between the medial frontal cortex and one cluster that included portions of the right postcentral and precentral gyri, and right central opercular cortex (Table 2E; Figure 3).

FE was associated with greater functional connectivity between the medial frontal cortex and two clusters (Figure 3). The first cluster included portions of the right middle and superior temporal gyrus, and the second cluster included portions of the right temporal pole and right orbitofrontal cortex.

## Discussion

This study aimed to identify differences in patterns of functional connectivity between older adults who self-reported a history of FE and older adults who reported no FE. Based on previous behavioral findings, and emerging models of FE risk in older adulthood, we targeted our analyses to regions implicated in decision-making and social cognition including the medial frontal cortex, hippocampus, and insular cortices. Our findings revealed differences in the hippocampal, insula, and medial frontal functional connectivity between FE and non-FE groups. These findings add and largely converge with the scant evidence linking brain changes to FE and FE-risk in normal aging (Han et al., 2016b; Spreng et al., 2017) and strengthen the case for identifying neural markers of FE risk.

We found differences in patterns of functional connectivity in bilateral hippocampal regions. Increased connectivity between the right hippocampus and regions of the left and right temporal lobe may reflect altered default network connectivity in older adults with a self-reported history of FE. Altered functioning of the default network has been implicated in a range of disorders, including Alzheimer’s disease (AD; Dickerson and Sperling, 2009), and recent work suggests that poor decision making and FE risk may be a very early sign of impending AD (Boyle et al., 2019; Stewart et al., 2019). It is well documented that functional neural changes associated with AD occur years before observed structural changes and clinical symptoms (Sperling et al., 2009; Cieri and Esposito, 2018). Some resting-state fMRI studies have reported increased functional connectivity between certain regions of the default network in participants at risk of developing AD, while others have reported decreased connectivity (for review Cieri and Esposito, 2018). Additionally, many task-based fMRI studies have found increased medial temporal lobe activity during episodic memory tasks in participants with mild cognitive impairment (MCI; e.g., Dickerson et al., 2005; Hämäläinen et al., 2007) suggesting possible compensatory responses to AD neuropathology (Dickerson and Sperling, 2009). Our findings of increased connectivity between the hippocampus and other regions in the FE group may reflect early brain changes associated with AD. However, our sample included only cognitively healthy older adults and thus, the present findings cannot confirm this possibility.

Further support for the relationship between FE risk and early AD comes from studies that have shown that older adults with mild cognitive impairment have poorer decision-making abilities (Griffith et al., 2003; Triebel et al., 2009; Han et al., 2015) and increased susceptibility to scams (Han et al., 2016a). A recent study by Boyle et al. (2019) demonstrated that low scam awareness, measured using a questionnaire, was associated with increased risk for AD and MCI, even after controlling for global cognitive function, in a community-based sample of older adults. The authors also found that low scam awareness was associated with a higher burden of Alzheimer’s pathology on autopsy. The authors suggest that decreased scam awareness in old age is an early sign of adverse cognitive outcomes, and in part a consequence of accumulating AD pathology. Future longitudinal studies will be useful in determining whether older adults reporting a history of FE are at greater risk of AD.

The hippocampus is also thought to play a role in prospection, or mental time travel (Buckner, 2010). Prospection is an important function for decision-making in that it allows an individual to envision the needs of the future with sufficient detail and predict possible outcomes of decisions to make an informed choice (Weierich et al., 2011; Lempert et al., 2020). Due to age-related changes to brain regions such as the medial temporal lobes important for prospection, Weierich et al. (2011) suggest that older adults have difficulty imagining the future in detail and are more likely to rely on affective states when making decisions. Thus, altered hippocampal connectivity may reflect dysfunctional prospection processes. This may place an individual at increased risk of FE by affecting the degree to which an individual considers the future consequences of a decision when confronted with an exploitative situation.

Findings of medial temporal lobe associations with FE are consistent with previous neuroimaging work from our group on susceptibility to scams (Han et al., 2016b; Lamar et al., 2019). In these studies, the lower structural integrity of the temporal lobes, and particularly the right medial temporal region (Han et al., 2016b), was associated with increased susceptibility to scams (measured using a six-item questionnaire). In the present study, the FE group showed altered functional connectivity between the hippocampus and regions within the temporal lobe, cerebellum, and occipital cortex. Since we know that medial temporal lobe regions like the hippocampus are susceptible to the effects of age-related neuropathology accumulation, FE risk in older adults may be a byproduct of early age-related pathological changes in the brain.

We also found greater connectivity in the FE group between the medial frontal cortex, the main hub of the default network (Raichle, 2015), and the right temporal regions. The only other study to our knowledge that investigated functional connectivity correlates of FE also found altered connectivity with the medial frontal cortex (Spreng et al., 2017), including reduced functional covariance between the precuneus and dorsomedial prefrontal cortex, thought to be part of the default network core (Andrews-Hanna et al., 2014). The authors did not report altered hippocampal connectivity, part of the dorsal medial subsystem of the default network (Andrews-Hanna et al., 2014; Buckner and DiNicola, 2019), as was found in this study. Methodological differences between studies may account for this difference. For example, while we took an ROI-based approach to identify differences in functional connectivity to specific brain regions, Spreng et al. utilized independent components analysis (ICA) to identify intrinsic networks and compared FE and non-FE groups on functional connectivity within the identified networks. Future research is needed to elucidate the specific patterns of altered connectivity within the default network and its subsystems in older adults who have experienced FE.

We found differences between FE and non-FE older adults in functional connectivity between brain regions important for decision making (hippocampus, medial frontal cortex) and motor and occipital regions. The nature of this finding is unclear. In a recent study by our group utilizing a larger sample which also included participants in the present study, we found that FE older adults reported greater symptoms of frailty, and specifically visual and hearing impairments, than non-FE older adults (Axelrod et al., in press). These findings may thus reflect a specific characteristic of our sample, and possibly vision and/or motor differences in FE and non-FE older adults. Future research may investigate the link between motor and sensory impairments and FE, and regions of the brain that underlie these functions.

FE older adults also demonstrated less functional connectivity between the right insula and the left lateral temporal lobes, including the left temporal pole, and middle and superior gyri. The implication of the role of the insula in FE risk converges with studies demonstrating its role in financial decision making (Samanez-Larkin et al., 2007; Samanez-Larkin and Knutson, 2015), and in particular in anticipating loss and gains (Samanez-Larkin et al., 2007). Research has demonstrated that older adults show comparable insular activation during anticipation of monetary gains but reduced activation during anticipation of monetary losses compared to young adults (Samanez-Larkin et al., 2007). The insula has also been implicated in assessments of trustworthiness (Winston et al., 2002; Castle et al., 2012), and studies of individuals with damage to their insular cortex have demonstrated abnormal expressions of interpersonal trust (Belfi et al., 2015). Thus, reduced insular connectivity with the lateral temporal lobes thought to play a role in socioemotional processing (Olson et al., 2013), belief attribution (Saxe et al., 2004), and theory of mind (Carrington and Bailey, 2009), may represent dysfunctional decision-making processes in social contexts, such as a tendency for FE older adults to believe that others are well-intentioned and trustworthy. This may impact decision making in social contexts, such as whether or not to believe a scammer. As suggested by Spreng et al. (2017), differences in insular connectivity may also impact the ability of older adults to detect threat in social contexts, given the insula’s role in salience-detection (Menon and Uddin, 2010), consequently leading to a greater risk of falling victim to exploitation.

Another possibility is that altered functional connectivity between the insula and regions of the temporal lobe may be a consequence of experiencing FE. Research supports the notion that the experience of psychosocial stress can change the underlying architecture of functional networks in the brain (Veer et al., 2011; Soares et al., 2013), and the experience of FE can result in significant distress to older adults (FINRA Investor Education Foundation, 2015). Along these lines, research suggests that the anterior insula marks risky social interactions so that individuals avoid similar interactions in the future (Rilling et al., 2008). Thus, altered functional connectivity between the insular cortex and temporal lobe regions may reflect a learned response following an exploitative social experience. With the current correlational methods, we cannot determine the direction of the effect of FE. Further work is necessary to determine risk factors for FE, as well as the functional consequences of FE on brain structure, function, and cognition.

Less connectivity was observed in the FE group compared to the non-FE group between both the right and left insula and the anterior and posterior cingulate cortex, and between the right insula and left dorsolateral prefrontal cortex. The cingulate cortex and dorsolateral prefrontal cortex are executive control regions that have been specifically implicated in cognitive control processes of decision making (Bush et al., 2002; Sanfey et al., 2003; Kennerley et al., 2006; Dixon and Christoff, 2014). The insula has been implicated in affective-based decision making (Sanfey et al., 2003; Bechara, 2004; Singer et al., 2009). Research supports the notion that decision making may depend on two interactive systems—an affective, emotion-driven system and a cognitive, deliberative system (Kahneman, 2003; Rolls, 2013; Okon-Singer et al., 2015). Thus, less connectivity between the insula and these control regions may reflect poor integration between cognitive and affective processes important for decision making. This lack of integration may increase the risk of FE, potentially through a tendency to make decisions largely based on emotional responses.

This study has several limitations that deserve mention. First, sample sizes of the FE and non-FE groups are small and this is a highly-educated sample; thus, findings are preliminary and should be cautiously interpreted and replicated to establish generalizability. Additionally, we utilized self-report to identify perceived FE and we did not objectively confirm FE experiences in the present study. This may introduce inaccuracies as the perception of FE may be inherently different than an experience of FE. Objective measures that evaluate the risk of FE (e.g., Lichtenberg et al., 2020) would be useful in validating group classification. The FE questions also did not allow for elaboration on the FE experience, including the type of FE experienced and the amount of money lost. These factors may affect the degree of functional connectivity differences observed between groups. Future studies may consider investigating how the specific features of an FE experience alter functional connectivity patterns. Another limitation is that the study is cross-sectional, and therefore causal links cannot be determined. The significant brain patterns that we observed might be consequences of FE and not precede FE. Longitudinal studies are needed to clarify the temporal nature of these associations. Finally, we utilized anatomical ROIs to investigate functional connectivity differences between groups. Other approaches to delineating functional connectivity metrics may yield different outcomes (Lv et al., 2018).

Nevertheless, this study is only the second to our knowledge that has investigated the functional connectivity correlates of FE. Findings of this study revealed differences in the hippocampal, insula, and medial frontal functional connectivity between FE and non-FE groups. These findings converge with the sparse evidence linking brain changes to FE and FE-risk in normal aging (Han et al., 2016b; Spreng et al., 2017). The present findings highlight potential differences in neural functional connectivity of older adults who believe they experienced FE, specifically in brain regions that have been identified as important for decision making in social contexts. A compelling implication of the present findings is that altered neural functional connectivity between brain regions important for decision making and social cognition may result in a vulnerability to FE in older adulthood ahead of any noticeable cognitive decline or impairment. Longitudinal studies are needed to examine this possibility.

## Data Availability Statement

The raw deidentified data supporting the conclusions of this article will be made available by the authors, without undue reservation, to qualified investigators upon request.

## Ethics Statement

The studies involving human participants were reviewed and approved by University of Southern California Institutional Review Board. The patients/participants provided their written informed consent to participate in this study.

## Author Contributions

GW: wrote the manuscript, analyzed the data, and assisted in data collection. LM: study conceptualization, review and editing of the manuscript. AN, JA, and CN: review and editing of the manuscript, assisted in data collection. PB and NS: review and editing of the manuscript. SDH: study conceptualization, writing, review, and editing of the manuscript. All authors contributed to the article and approved the submitted version.

## Conflict of Interest

The authors declare that the research was conducted in the absence of any commercial or financial relationships that could be construed as a potential conflict of interest.

## Footnotes

^1^https://web.conn-toolbox.org/home

^2^https://www.nitrc.org/projects/artifact_detect/
